# Caloric restriction impacts skin barrier function and attenuates the development of hyperplasia skin disease

**DOI:** 10.3389/fnut.2024.1423524

**Published:** 2024-09-20

**Authors:** Huihao Tang, Jianzhou Li, Mengyu Jin, Chengliang Li, Chuntao Zhai, Juan Wang, Taomin Huang, Xiaolei Ding

**Affiliations:** ^1^Institute of Geriatrics, Affiliated Nantong Hospital of Shanghai University (The Sixth People's Hospital of Nantong), School of Medicine, Shanghai University, Nantong, China; ^2^Shanghai Engineering Research Center of Organ Repair, School of Medicine, Shanghai University, Shanghai, China; ^3^Joint International Research Laboratory of Biomaterials and Biotechnology in Organ Repair (Ministry of Education), Shanghai University, Shanghai, China; ^4^LB Cosmeceutical Technology Co., Ltd., Shanghai, China; ^5^Department of Pharmacy, Eye & ENT Hospital, Fudan University, Shanghai, China; ^6^Department of Dermatology, Huashan Hospital, Fudan University, Shanghai, China

**Keywords:** diet, caloric restriction, skin barrier function, inflammatory response, mTOR

## Abstract

Caloric restriction (CR) stands out as one of the most potent interventions that prolong lifespan and mitigate age-associated diseases. Despite its well-established systemic effects, the impact of CR on skin physiological function remains poorly understood, and whether the intervention can alleviate the progression of inflammatory skin diseases remains uncertain. Here, we investigated the effects of CR on mouse skin barrier function and inflammatory response. Our results revealed that CR led to dramatic atrophy in the skin subcutaneous layer. The expression of barrier proteins and trans-epidermal water loss remain largely unchanged. Intriguingly, skin from CR mice exhibited reduced expression of inflammatory cytokines under steady conditions. In an imiquimod (IMQ)-induced mouse model of psoriasis, CR treatment attenuated the pathogenesis of psoriasis phenotypes, accompanied by a reduced activation of mTOR signaling in the psoriatic skin. Taken together, our findings shed light on the complex interplay between metabolic interventions and skin health, suggesting that CR has the potential to serve as a modulator of inflammatory responses in the skin.

## 1 Introduction

Diet plays a pivotal role in providing essential nutrients and profoundly influences overall health and disease progression. Among dietary interventions, caloric restriction (CR) has garnered considerable attention due to its potential health benefits, including extending lifespan and reducing the incidence of age-associated diseases such as metabolic disorders, kidney damage, tumors, and cardiovascular disorders (1–4). These beneficial effects are thought to be mediated by diverse mechanisms, including alleviating oxidative stress, increasing insulin sensitivity, regulating neuroendocrine responses, reducing necrosis, and delaying tumor development (3, 4). Moreover, restricted diets or periodic fasting have been shown to improve inflammation by affecting immune cell metabolism, leading to reversal of abnormal changes in immune cell proportions (5, 6). However, recent studies have highlighted potential adverse effects of inadequate caloric intake on tissue structure and function. For example, low caloric intake has been associated with endothelial dysfunction and increased susceptibility to cardiac arrhythmias and pathology in male rats (7, 8), and female rat heart appears to exhibit increased vulnerability to the long term adverse cardiovascular effects of severe food restriction (9). Additionally, while fasting may initially reduce circulating monocyte numbers, refeeding can trigger their release into the bloodstream, potentially heightening susceptibility to infections (10). These findings underscore the importance of carefully evaluating the effects of CR on specific tissues and organs, particularly in the context of infections and disease development. Further research is warranted to comprehensively understand the potential benefits and risks of CR, as well as the optimal strategies for its implementation in different settings.

The skin, a highly intricate organ situated at the interface between the organism and the external environment, serves as a crucial barrier against a myriad of external threats, including pathogens, chemicals, and physical trauma. Recent advances in our understanding of skin biology have revealed that the maintenance of skin structural and functional integrity is not solely governed by local factors but also is influenced by systemic conditions, such as nutritional status (11, 12). Excessive calorie intake, characterized by the Western diet (WD), has been implicated in various skin disorders (2, 13). Studies indicate that mice fed with the WD exhibit compromised skin barrier function and increased immune activation (14). Obesity is considered a risk factor for the development of various inflammatory diseases, including psoriasis, a chronic inflammatory skin disease characterized by enhanced proliferation and differentiation of the epidermis (15). High-fat diet-induced obesity can exacerbate imiquimod (IMQ)-induced psoriatic dermatitis in mice (16). By contrast, dietary intervention, particularly with saturated free fatty acid reduction can reduce disease progress and general inflammatory status in mouse models and psoriasis patients (17, 18). Moreover, investigations using mice as model organisms have demonstrated the potential benefits of CR on skin health. For instance, food intake restriction in mice has been shown to promote extensive skin and fur removal, contributing to thermal homeostasis and metabolic fitness (19). Additionally, CR has been shown to increase epidermal thickness in female hairless mice exposed to long-term UVB irradiation, thereby mitigating hidradenitis suppurativa severity and other dermatoses (20, 21). Despite these findings, whether and how CR affects skin barrier function remain unclear.

In this study, we aim to explore the consequences of short-term CR on skin homeostasis. Our findings revealed that CR could regulate skin barrier function with a notable reduction in inflammatory cytokines expression. Using an IMQ-induced psoriatic model, we found that CR could attenuate the psoriatic pathogenesis, which was accompanied by a reduction of mTOR signaling activation. These results provide valuable mechanistic insights into the intricate relationship between dietary interventions and skin health, offering potential avenues for the development of innovative dermatological treatments and protective strategies.

## 2 Materials and methods

### 2.1 Mice and CR administration

All experiments were performed with female 8-week-old BALB/c mice with a body weight ranging from 18 to 22 g. The mice were obtained from Chuang Hua (China) and maintained under standard pathogen-free conditions. Body weight and age-matched mice were randomly divided into either the *ad libitum* (AL) -fed or CR-fed group. One week before the dietary intervention, mice were individually housed and their daily food consumption was measured for every mouse to determine their AL-feeding rate. The average amount of food was determined after the 1-week measurement for every mouse. CR mice were fed daily with an amount of food corresponding to 70% of that consumed by body weight-and gender-matched mice in the AL group as previously described (22–26). Upon initiating the feeding protocol, the AL mice were fed with unlimited access to food, while CR mice were fed with 70% of the average amount of food according to the previous calculation. The food pellet was added to each cage daily at the same time, and remained constant over the whole CR period (24). All experiments were approved according to the Animal Experimentation Ethics Committee of Shanghai University.

### 2.2 IMQ-induced psoriasis model

Female BALB/c mice (8 weeks of age; 18–22 g) were randomly divided into 4 groups: (1) the AL + Vaseline group; (2) the AL + IMQ group; (3) the CR + Vaseline group; and (4) the CR + IMQ group. Female mice were used because females are generally more sensitive to IMQ than males (27). A daily topical dose of 62.5 mg IMQ cream containing 5% IMQ (Medicine Shine, China) was applied to a shaved area (3 × 2.5 cm) on the mice’s dorsal skin for 5 consecutive days, to create a psoriasis-like mouse model as previously described (28–30). Vaseline was used as a control. On day 5, full-thickness skin biopsies of the treated area were collected for further experiments. The skin was fixed in paraformaldehyde, or Optimal Cutting Temperature compound (OCT) for histopathological analysis and was excised for RNA preparation (31).

### 2.3 Physiological parameter measurement

Body weight and blood glucose levels were monitored weekly. Ear thickness was measured weekly using a digital caliper. Trans-Epidermal Water Loss (TWEL) was assessed with the gpskin Barrier Light measurement tool (gpower, Seoul).

### 2.4 Psoriasis area severity index analysis

The severity of psoriatic lesions on the dorsal skin was evaluated using the Psoriasis Area Severity Index (PASI). Clinical observation was conducted daily. The PASI assessment includes evaluating the area of dorsal skin lesions, erythema, scaling, and thickening for 5 consecutive days. The PASI scores are 4 grades, including 0 (none); 1 (light); 2 (moderate); 3 (severe); and 4 (extremely severe) (32).

### 2.5 RNA isolation and quantitative real-time PCR analysis

Total RNA was extracted from skin samples using TRIzol (Invitrogen) according to the manufactures’ protocol. The concentration of RNA was measured using an ultraviolet spectrophotometer (Beckman, DU-530). Reverse transcription of the isolated RNA was performed using the PrimeScript RT Reagent Kit (Takara) according to the manufactures’ protocol. Amplification reactions were performed on a fluorescence quantitative polymerase chain reaction (PCR) instrument (ViiA7, ThermoFisher Scientific) using the SYBR Premix Ex Taq II formulation (Takar). The sequence information of primers used in this study are listed in Table 1. The relative mRNA levels were determined by the 2^−ΔΔCt^ method, normalized to the GAPDH as a housekeeping gene.

**Table 1:** Sequence of primers.

Name	Forward	Reverse
*Loricrin*	TCACTCATCTTCCCTGGTGCTT	GTCTTTCCACAACCCACAGGA
*Filaggrin*	GGAGGCATGGTGGAACTGA	TGTTTATCTTTTCCCTCACTTCTACATC
*Involucrin*	TCCTCCAGTCAATACCCATCAG	CAGCAGTCATGTGCTTTTCCT
*Keratin10*	CGTACTGTTCAGGGTCTGGAG	GCTTCCAGCGATTGTTTCA
*Keratin 14*	TCGATCTGCAGGAGGACATT	ATCGAGGACCTGAAGAGCAA
*Vegf-a*	CCTGGCCCTCAAGTACACCTT	TCCGTACGACGCATTTCTAG
*IFN-γ*	TAACTCAAGTGGCATAGATGTGGAAG	GACGCTTATGTTGTTGCTGATGG
*IL-6*	ACACATGTTCTCTGGGAAATC	AAGTGCATCATCGTTGTTCATACA
*TNF-α*	AGTGACAAGCCTGTAGCCC	GAGGTTGACTTTCTCCTGGTAT
*IL-1β*	GGACCCCAAAAGATGAAGGGCTGC	GCTCTTGTTGATGTGCTGCTGCG
*IL-17A*	GCCCAGTCTCTTTGTGTTAG	CTG ATTATGTTTGTTTTCTTTCC
*IL-23A*	CCCGTATCCAGTGTGAAG	GATGTCAGAGTCAAGCAGGT
*IL-2*	TTGGACCCTGGCACCTACAATG	GCAGACAGGCTTTGCAGAATGG
*IL-18*	TCAGAAGACTCTTGCGTCAA	CCGTATTACTGCGGTTGT
*GAPDH*	AGGTCGGTGTGAACGGATTTG	TGTAGACCATGTAGTTGAGGTCA

### 2.6 Immunohistochemistry and immunofluorescence staining

Mouse back skin was collected and either fixed in 4% paraformaldehyde or embedded in OCT compound (OCT, Tissue Tek). Embedded tissues were sectioned (4 μm or 10 μm). For histological analysis, sections were stained with hematoxylin and eosin (H&E) staining. Immunohistochemistry and immunofluorescent staining were performed as described previously (33, 34). Briefly, antigen was retrieved using citric acid buffer (pH = 6.0). Tissues were permeabilized in 0.5% Triton with PBS for 10 min at RT, and blocked with 100% normal goat serum in PBS for 1 h at RT. Then, the sections were incubated with indicated primary antibodies overnight at 4°C. The primary antibodies were detected using corresponding second antibodies. For immunofluorescence staining, 4′,6-diamidino-2-phenylindole (DAPI) (Solarbio) was used to visualize the nuclei. For immunohistochemistry staining, the signal was detected with 3,3¢-diaminobenzidine (DAB) staining, followed by counterstaining with hematoxylin. Negative control sections were treated in the same way, omitting the primary antibody. The following antibodies against specific antigens were used: Ki-67, K14, and CD3 (all from Abcam, ab15580, ab181595 and ab16669), CD31 (BD, 557355), Loricrin (Invitrogen, PA5-30583), K10 (Santa Curze, SC-23877), Filaggrin (Biolegend, #905804), CD68, p-S6 and p-4EBP1 (all from Cell Signaling Technology: #97778, #5364 s and #2855); IL-17RA (1:100; bs-2606R, Bioss Antibodies); IL-17A (1:100, 26,163-1-AP, proteintech); (HRP)-conjugated goat anti-rabbit IgG (IH-0001, Guo Ding, China); anti-Rabbit Alexa Fluor488 (Invitrogen, A-11008); anti-mouse Alexa Fluor594 (Invitrogen, A11005), anti-Rat Alexa Fluor594 (Invitrogen, A-11007). Images were taken using the Olympus Microscope IX71 (Olympus, Tokyo, Japan). Five visions were counted per tissue section used for measurement. The mean fluorescence intensity and cell quantity of skin was quantified from photographs using ImageJ software with the IHC Toolbox.

### 2.7 Statistical analysis

All data were represented as mean ± SEM. Statistical analysis was performed using One-way or Two-way ANOVA with Tukey’s correction for multiple comparisons. GraphPad Prism software was utilized for data analysis. Statistical significance was considered at *p < 0.05.

## 3 Results

### 3.1 CR leads to hypodermis atrophy

**Figure 1:**
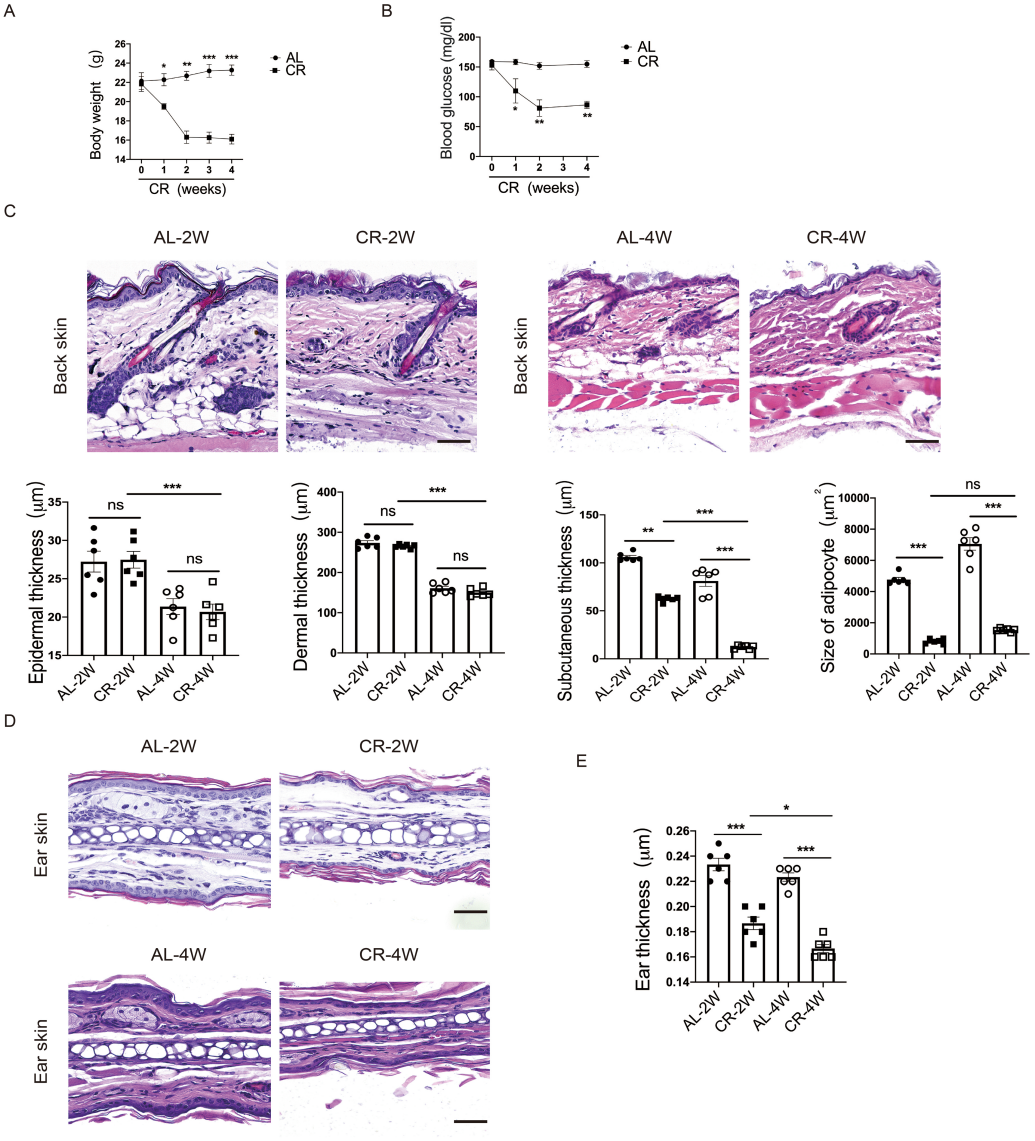
CR leads to mouse skin atrophy. **(A)** Body weights of CR-fed and AL-fed mice over 4 weeks. **(B)** Blood glucose levels of CR-fed and AL-fed mice over 4 weeks. **(C)** Representative images of H&E stained back-skin sections of the AL-and CR-fed mice at indicated time. Scale bar: 100 μm. Quantification of skin epidermis, dermis and subcutaneous layer thickness, as well as the size of dermal white adipocytes. **(D)** Representative images of H&E-stained ear tissue sections. Scale bar: 100 μm. **(E)** The measurement of ear thickness of the AL- and CR-fed mice. Each dot represents one mouse. Data are presented as mean ± SEM (*n* = 6), and statistical significance was determined by Two-way ANOVA for multiple comparisons. * *p* < 0.05, ** *p* < 0.01, *** *p* < 0.001; ns, not significant.

To assess the effects of systemic CR on skin structure and barrier function, we subjected 8-week-old female BALB/c mice on CR by limiting their daily food availability to 70% for 2 and 4 weeks. Throughout the study, the body weights of mice were monitored weekly. Compared to AL-fed control mice, the body weights of mice on CR were progressively declined, along with the reduced levels of blood glucose (Figures 1A,B). Importantly, CR did not induce macroscopic abnormalities or skin lesions, consistent with observations of previous studies (35).

To evaluate the effect of CR on skin structure, we conducted histological analysis of back skin tissue from CR-fed mice and AL-fed control mice. H&E-stained paraffin sections revealed no significant damage or disruption of the epidermal structure compared to AL-fed control skins. Similarly, CR did not induce apparent alterations in the dermal compartment. However, the thickness of skin subcutaneous layer was significantly reduced in CR-fed mouse skin. Furthermore, the size of adipocytes in the subcutaneous layer was significantly diminished in CR-fed mice compared to AL-fed mice, indicative of reduced fat volume (Figure 1C). Additionally, ear thickness was also visibly decreased compared with AL-fed mice (Figures 1D,E), indicating that CR led to skin atrophy, which is primarily attributed to decreased cell volumes of subcutaneous adipocyte.

### 3.2 CR impacts on skin barrier function

**Figure 2:**
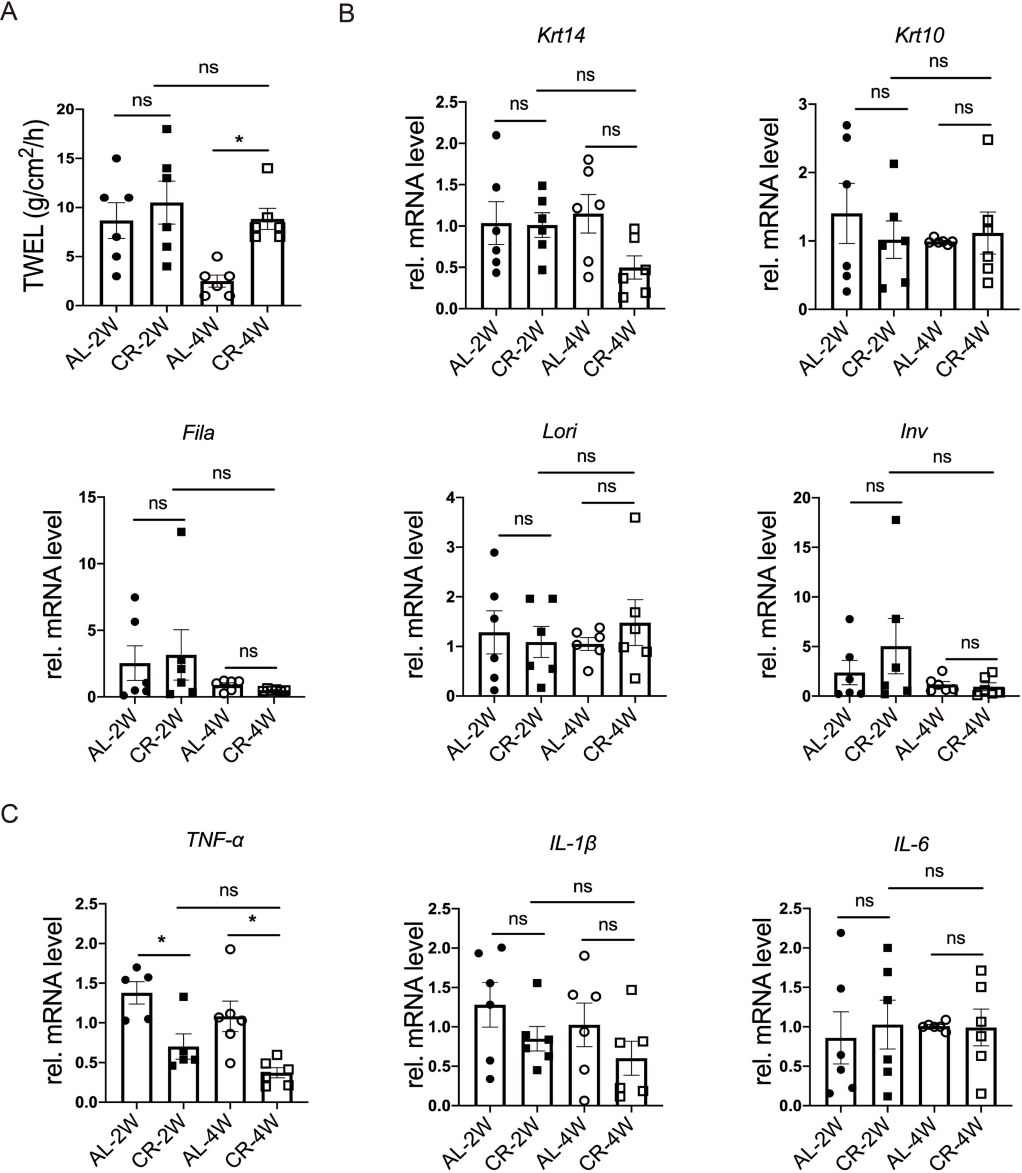
CR impacts on skin barrier function. (A) Quantification of TEWL of CR-fed and AL-fed mice. **(B)** RT-PCR analysis of mRNA expression levels of Krt14, Krt10, Lori, Fila and Inv. **(C)** The determination of relatively mRNA expression levels of TNF-*α*, IL-1β and IL-6. mRNA expression of one sample from AL-2 W group was set as 1. Each dot represents one mouse. Data are presented as mean ± SEM (*n* = 5 ~ 6), and statistical significance was determined by Two-way ANOVA for multiple comparisons. * *p* < 0.05; ns, not significant.

Trans-epidermal water loss (TEWL) measures the amount of water loss through the skin, which diffuses from inside the body across the stratum corneum. To evaluate the impact of CR on skin barrier function, we measured TEWL in mice at 2 weeks and 4 weeks following CR initiation. After 2 weeks of CR, no significant difference in TEWL was observed between CR-fed and AL-fed mice. However, at 4 weeks of CR, TEWL was slightly increased compared to AL-fed mice (Figure 2A), indicating a potential impairment of the epidermal barrier function with prolonged CR. These results suggested that short-term CR may not significantly affect skin barrier function, but prolonged CR could lead to increased water loss through the skin.

To investigate how CR impacts skin barrier homeostasis, we isolated RNA and measured the mRNA levels of epidermal stratification makers, including keratin 14 (Krt14), keratin 10 (Krt10), as well as representative barrier proteins, such as loricrin (Lori), filaggrin (Fila), involucrin (Inv) by using RT-PCR (36, 37). At both 2 and 4 weeks of CR, the RNA levels of *Krt10, Lori, Fila, Inv* were comparable between CR-fed and Al-fed mouse skin tissues (Figure 2B), providing further evidence that short-term CR did not affect the barrier protein expression in the epidermis.

Skin barrier disruption can affect the expression of inflammatory cytokines in the skin (38, 39). To further characterize the response of CR to barrier impairment, we measured the relative mRNA levels of *TNF-α*, *IL-1β* and *IL-6*, typical inflammatory markers, using RT-PCR. Our results demonstrated that the mRNA level of *TNF-α* was markedly decreased at 2 or 4 weeks compared with the AL-fed group (Figure 2C). And the mRNA levels of *IL-1β* and *IL-6* were unchanged at 2 or 4 weeks compared with the AL-fed group (Figure 2C). The lacking exaggerated but reduced expression of inflammatory cytokines indicates that CR causes no major disruption of skin barrier, particularly the outside-in function. These results suggested that short-term CR reduces skin inflammation under steady-state conditions and cause rarely barrier disruption.

### 3.3 CR inhibits the development of epidermal hyperplasia

**Figure 3:**
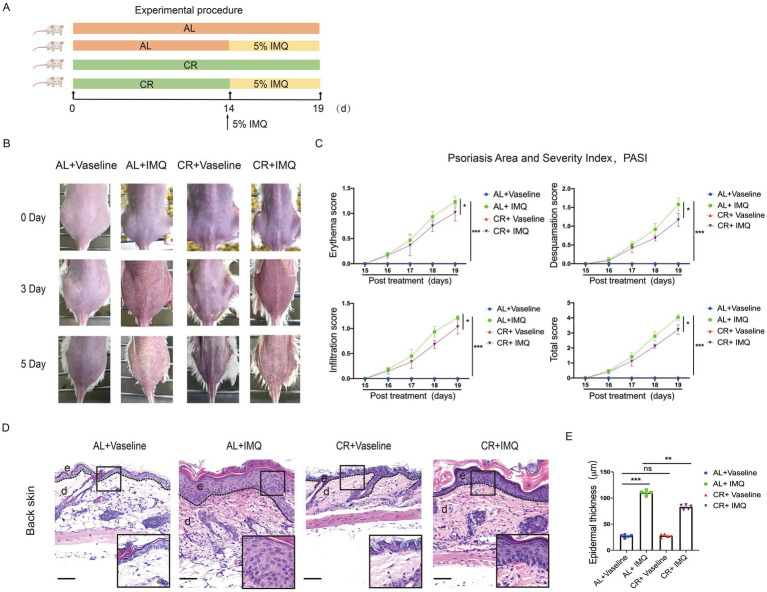
CR delays skin symptoms of IMQ-induced Psoriasis. **(A)** Experimental scheme. **(B)** The back skin of mice in each group on different days. **(C)** PASI scores (Erythema, Desquamation, Infiltration) of skin lesions in each group of mice were scored daily. **(D)** Left, histopathological changes of skin lesions were determined by H&E staining. Scale bar: 100 μm. **(E)** The measurement of epidermal thickness of mice’s back skin. Each dot represents one mouse. Data are presented as mean ± SEM (*n* = 5), and statistical significance was determined by two-way ANOVA for multiple comparisons. * *p* < 0.05, ** *p* < 0.01, *** *p* < 0.001; ns, not significant; e, epidermis; d, dermis.

To investigate the effects of CR on the development of hyperplasia skin disease, we used an IMQ-induced mouse model which is known for inducing psoriasis-like lesions via Toll-like receptor (TLR)7/8 agonist stimulation (40). Given that 4 weeks of CR led to impaired skin barrier function, we initiated IMQ application after 2 weeks of CR to mitigate potential confounding effects. As illustrated in the Figure 3A, BALB/c mice were subjected to either CR or AL diets for 2 weeks prior to the experiment. Subsequently, IMQ or Vaseline was applied to the shaved areas on mouse back skin for 5 consecutive days. Three days post IMQ application, signs of skin thickening and erythema manifested on the dorsal skin of the mice (Figure 3B). The characteristic features of IMQ-induced skin inflammation, including erythema, desquamation, and cellular infiltration, were assessed daily throughout the experiment (41). Both individual and total scores exhibited significant reductions in IMQ-treated CR-fed mice, compared to IMQ-treated AL-fed control mice (Figure 3C). Histological examination using H&E staining revealed distinct histopathological features in the dorsal back skin of mice in the IMQ-treated AL-fed control group, characterized by a thickened epidermal spinous cell layer, parakeratosis, and extensive inflammatory cell infiltration within the dermal layers (Figure 3D). In contrast, in the IMQ-treated CR-fed mice, the observed symptoms were alleviated, evidenced by a reduced number of parakeratotic cells in the dorsal skin lesions, a thinner epidermal spinous cell layer, and a decrease in epidermal thickness (Figures 3D,E). These results suggested that short-term CR can attenuate the development of psoriasis-like skin phenotype.

### 3.4 CR attenuates cell proliferation in the psoriasis skin

**Figure 4:**
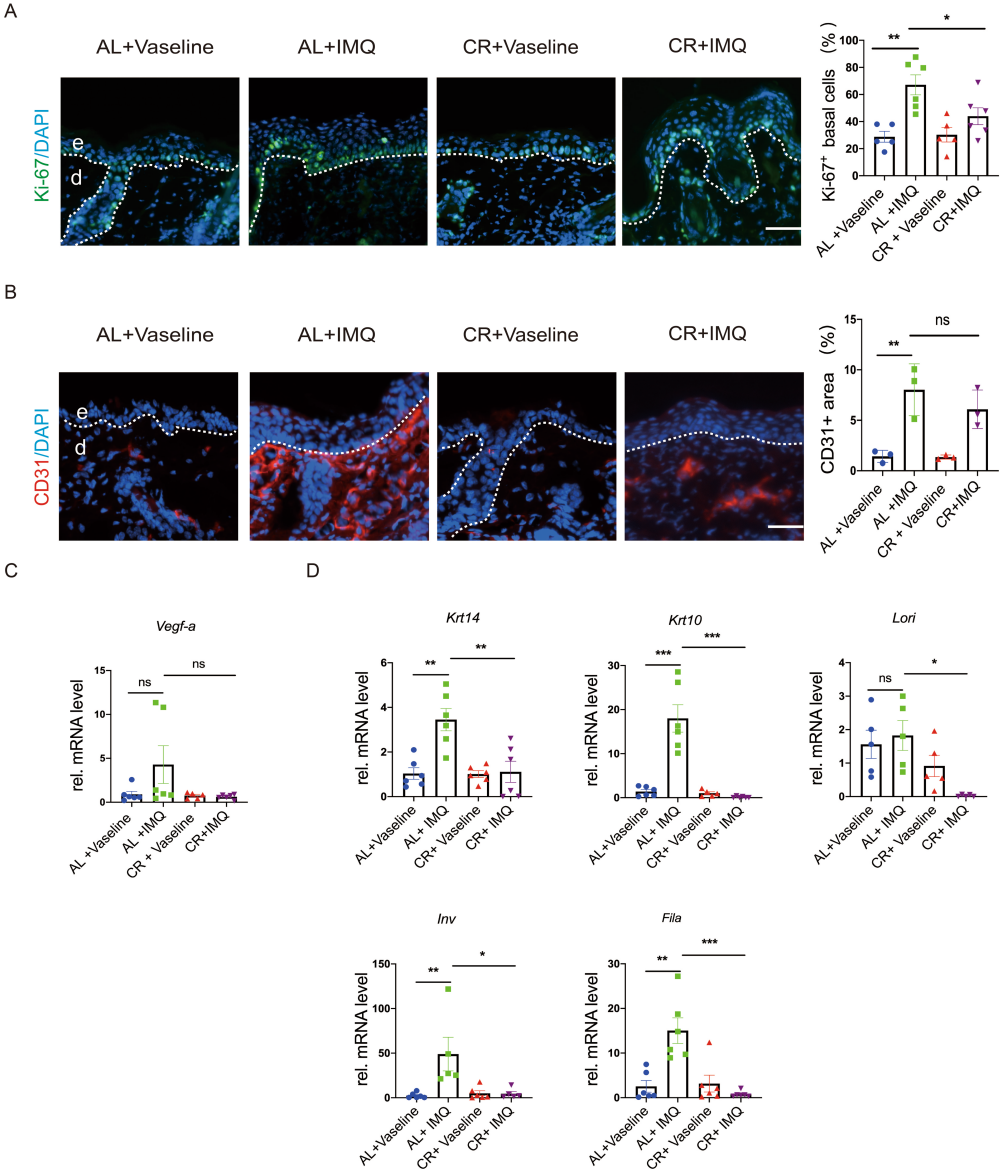
CR reduces cell proliferation in IMQ-induced epidermal hyperplasia. **(A)** Left, Ki-67 staining of skin lesions by the immunofluorescence assay. Scale bar: 50 μm. Right, the percentage of Ki-67+ cells in basal layer cells in the skin lesions (*n* = 5). **(B)** Left, CD31 staining of skin lesions by the immunofluorescence assay. Right, the mean fluorescence intensity of CD31expression in the skin lesions (*n* = 3). **(C)** The determination of mRNA expression levels of *Vegf-a* related to angiogenesis. **(D)** The determination of mRNA expression levels of *Krt14, Krt10, Lori, Fila* and *Inv* related to skin epidermal barrier and differentiation. Each dot represents one mouse. mRNA expression of one sample from AL + Vaseline group was set as 1. Scale bar: 50 μm. Data are presented as mean ± SEM (*n* = 3 ~ 6), and statistical significance was determined by two-way for multiple comparisons. * *p* < 0.05, *** *p* < 0.001; ns, not significant; e, epidermis; d, dermis.

To examine whether the decreased epidermal thickness observed in IMQ-treated CR-fed mice resulted from a reduction in cell proliferation, we evaluated the expression of Ki-67, a cell proliferation marker, in skin tissues by using immunostaining. Ki-67-positive cells were predominantly located in the epidermal basal layer and covered most of the basal layer in IMQ-treated AL-fed mice compared with the Vaseline-treated AL-fed mice, indicating a hyperproliferative response to IMQ treatment. However, the number of Ki-67 positive cells was significantly reduced in IMQ-treated CR-fed mouse skin compared with IMQ-treated AL-fed group (Figure 4A). Furthermore, we detected the expression of CD31, a marker for angiogenesis by the immunofluorescent staining. The relative mRNA level of *Vegf-a* (vascular endothelial growth factor a) was also examined. As expected, the expression of CD31 and *Vegf-a* was markedly increased in skin lesions by IMQ-treated. IMQ-treated CR-fed mouse skin exhibited a trend towards reducing CD31 and *Vegf-a* expression, compared to the IMQ-treated AL-fed control mice. However, the significant difference in statistics was not reached (Figures 4B,C).

To assess whether CR influences epidermal barrier in the skin of mice with IMQ-treated psoriasis, we examined the mRNA expression levels of *Krt14, Krt10, Lori, Fila* and *Inv* by RT-PCR. Our results showed that the relative mRNA levels of *Krt14, Krt10, Fila* and *Inv* were significantly upregulated in the IMQ-treated AL-fed group (Figure 4D), largely consistent with previous reviews (41). Consistently, barrier regulators, like K14, K10, Filaggrin, Loricrin and showed similar alterations at protein levels in the skin lesions, as revealed by immunostaining (Supplementary Figure S1). This upregulation was notably inhibited in IMQ-treated CR-fed mice, indicating that CR may not only reduce keratinocyte proliferation, but also potentially inhibit keratinocyte differentiation under pathological condition.

### 3.5 CR decreases the inflammatory responses in the IMQ-treated psoriatic model

**Figure 5:**
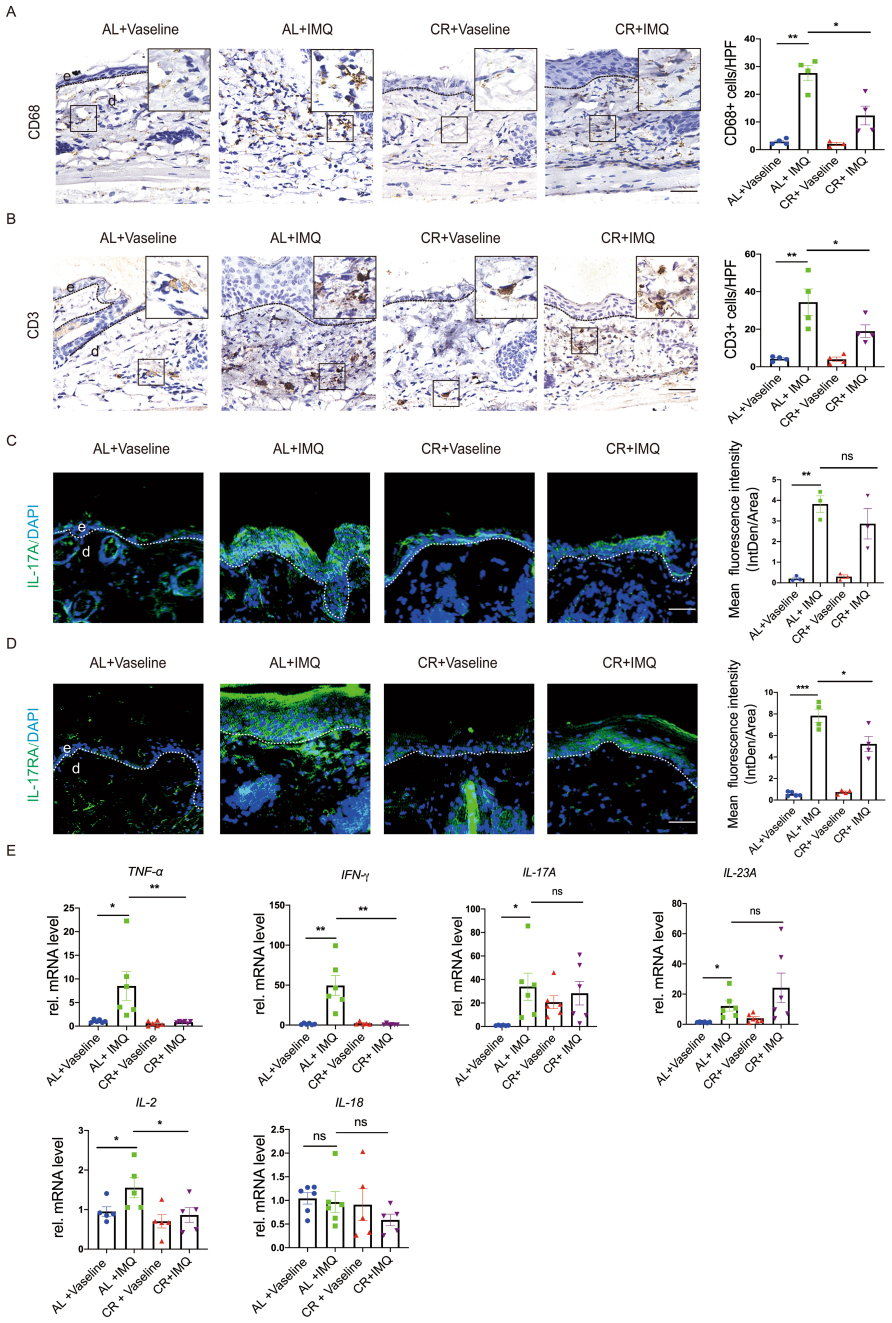
CR reduces inflammatory responses in IMQ-treated skin lesions. **(A)** Left, representative images of CD68+ immunohistochemistry staining of skin tissues. Scale bar: 50 μm. Right, the quantitative analysis of CD68+ cells in skin dermal lesions. **(B)** Left, representative images of CD3+ immunohistochemistry staining of skin tissues. Scale bar: 50 μm. Right, the quantitative analysis of CD3+ cells in skin dermal lesions. **(C)** Left, IL-17A staining of skin lesions by the immunofluorescence assay. Right, the mean fluorescence intensity of IL-17A expression in the skin epidermal lesions (*n* = 3). Scale bar: 100 μm. **(D)** Left, IL-17RA staining of skin lesions by the immunofluorescence assay. Right, the mean fluorescence intensity of IL-17RA expression in the skin epidermal lesions (*n* = 4 ~ 5). Scale bar: 100 μm. **(E)** Analysis of *TNF-α*, *IFN-γ*, *IL-17A*, *IL-23A*, *IL-2* and *IL-18* gene expression levels in skin tissues. mRNA expression of one sample from AL + Vaseline group was set as 1. Each dot represents one mouse. Data are presented as mean ± SEM (*n* = 5 ~ 6), and statistical significance was determined by two-way ANOVA for multiple comparisons. * *p* < 0.05, ** *p* < 0.01, *** *p* < 0.001; ns, not significant; e, epidermis; d, dermis. HPF, high power field.

Inflammation is a major driver of psoriasis development, primarily associated with dermal leukocyte infiltration and the release of pro-inflammatory cytokines (42, 43). CR has been shown to intricately regulate the inflammatory cell infiltration in the body (3, 4). To evaluate whether the changes in the disease development between IMQ-treated CR-fed and AL-fed mice skin correlated with inflammatory responses, lesional skin tissues were stained for the macrophage maker CD68 and T cell maker CD3. Immunostaining of CD68 revealed a significant increase in the presence of macrophages in the skin of IMQ-treated AL-fed mice compared with Vaseline-treated control skin, indicating enhanced immune response. In the skin of IMQ-treated CR-fed mice, the number of macrophages was significantly reduced compared with the skin of IMQ-treated CR-fed mice. CD3 staining and the quantitative evaluation revealed the accumulation of T cells was also reduced in the skin of IMQ-treated CR-fed mice compared with IMQ-treated AL-fed mice skin. These results indicated that the protected hyperplasia skin disease development by short-term CR correlated with the decreased immune cells infiltration in the dermis (Figure 5A).

To further determine the inflammatory changes, we examined the expression of inflammatory cytokines by using RT-PCR. Our results revealed a significant increase in the mRNA levels of inflammatory cytokines, such as *TNF-α*, *IFN-γ*, *IL-17A*, *IL-23A*, *IL-2* and *IL-18* in the IMQ-treated AL-fed mice, compared with Vaseline-treated AL-fed mouse group (Figure 5B), similar with previous investigation (44). This up-regulation of *TNF-α* and *IFN-γ* expression was not observed in IMQ-treated CR-fed mouse skin. *IL-17A* mRNA level was comparable between IMQ-treated CR-fed mouse skin and AL-fed control group. Unexpectedly, *IL-23A* mRNA expression showed a trend of increasement in IMQ-treated CR-fed mouse skin, compared to IMQ-treated AL-fed mouse skin. *IL-2* mRNA level was slightly decreased in IMQ-treated CR-fed mouse skin, compared to IMQ-treated AL-fed mouse skin. *IL-18* mRNA level was slightly decreased in IMQ-treated CR-fed mouse skin, compared to IMQ-treated AL-fed mouse skin, but it had no significance (Figure 5E). We also performed immunostaining of IL-17A and its receptor, IL-17RA and in the lesional skin tissue. The results showed that the levels of IL-17A and IL-17RA expression were significantly increased in the IMQ-treated AL-fed group, compared to the controls. CR reduced IMQ-induced IL-17A and IL-17RA expression in the skin (Figures 5C,D). Collectively, these results suggested that CR inhibits skin inflammatory responses under pathological conditions, which may be involved in different immune cell systems.

### 3.6 CR attenuates the activation of mTOR signaling in psoriatic skin

**Figure 6:**
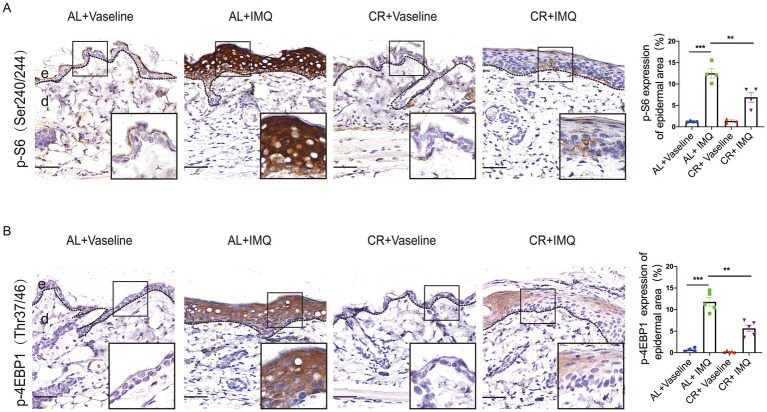
The attenuated activation of mTORC1 singling in psoriatic skin from CR mice. **(A)** p-S6 staining of skin lesions by immunohistochemistry staining in the skin epidermal lesions. Scale bar: 50 μm. The quantitative analysis of p-S6 expression in skin epidermal lesions (*n* = 3 ~ 4). **(B)** p-4E-BP1 staining of skin lesions by immunohistochemistry staining in the skin epidermal lesions. Scale bar: 50 μm. The quantitative analysis of p-4E-BP1 expression in skin epidermal lesions (*n* = 4 ~ 5), and statistical significance was determined by two-way for multiple comparisons. ** *p* < 0.01, and *** *p* < 0.001. e, epidermis; d, dermis.

CR action largely depends on the modulation of nutrient-sensing pathways, particularly the mammalian target of rapamycin complex 1 (mTORC1), which orchestrates cellular responses to nutrient availability and metabolic cues and has been implicated in the development of human psoriatic disease (45–48). We therefore tested whether CR was able to interfere with the activation of mTOR signaling pathway. We conducted immunohistochemistry of phosphorylated-S6 and 4E-BP1, well-characterized downstream effectors of mTORC1. While, normal skin showed hardly any phosphorylation of S6 at ser240/244 and 4E-BP1, IMQ treatment induced a significant increase in phosphorylation at in the epidermis, particular the basal cells. These findings are consistent with previous studies (49), indicating that the concurrent activation of mTORC1 signaling is concurrent with psoriasis development (Figure 6A). Strikingly, compared with IMQ-treated AL-fed mice, phosphorylated S6 and 4E-BP1 were only mildly increased in IMQ-treated CR-fed mouse skin tissues (Figure 6B), suggesting that CR could dampen the activation of mTORC1 signaling during psoriasis development. Our data indicated that CR ameliorated mTOR signaling activation under pathological conditions, implying that the IMQ-induced psoriatic phenotype might be through mTOR signaling inhibition.

## 4 Discussion

In this study, we examined the effects of CR on skin barrier integrity and preventing the development of hyperplasic skin disorders. Our findings revealed that CR significantly decreased subcutaneous tissue thickness. Short-term CR did not show evident defects in skin barrier function. The expression of inflammatory cytokine was reduced in the skin of CR mice. Moreover, CR mitigated IMQ-induced skin psoriatic phenotypes, possibly through inhibiting the mTOR signaling pathway, a central regulator of cell growth. These results suggest that short-term CR or modulating food intake may serve as a potentially beneficial approach for regulating skin hyperplasia diseases.

The skin barrier function is essential for protecting against water loss and the penetration of irritants. The outermost layer stratified epidermis, known as the stratum corneum, plays a critical role in this primary barrier function. Key components regulating skin barrier function, including barrier proteins and lipids have been identified. These components are interdependent, and proper functioning of each factor is necessary for maintaining the integrity of the skin barrier. Our findings indicated that the expression of barrier components remained largely unaffected upon CR. TEWL remained unchanged after 2 weeks of CR, indicating that short-term CR may induce structural changes in the skin without compromising its barrier integrity. We observed that TEWL was slightly increased in mice at 4 weeks with CR, implying a possible negative effect of prolonged CR. Of note, we employed a protocol of CR by directly reducing food intake (26). It is crucial to determine a long-term and sustained CR regimen without malnutrition, and it is warranted to confirm these findings, providing the critical information about safety and feasibility of CR in human (14).

The beneficial effects of CR on systemic low-grade inflammation and metabolism and are widely recognized. A noteworthy observation from our study is the reduced expression of inflammatory cytokines in the skin of CR mice. This downregulation of inflammatory mediators suggests a potential anti-inflammatory effect of CR on the skin, which is consistent with findings from studies investigating the anti-inflammatory effects of CR in other tissues and organs and may contribute to its protective effects against inflammatory skin diseases (50). The modulation of inflammatory responses by CR highlights its broader impact on immune regulation and tissue homeostasis, which could have significant implications for age-related skin conditions and inflammatory skin diseases.

Skin is a dynamically active tissue, exhibiting a remarkable ability to adapt its size and function in response to diverse internal and external stimuli. Our research elucidated substantial histological alterations in the subcutaneous layer of mice subjected to CR, highlighting the profound influence of CR on skin morphology. Subcutaneous layer mainly consists of dermal adipose tissue (dWAT), which plays a key role in providing resilience to external stress factors and has attracted attentions of research. Recent studies, including our own, have demonstrated the remarkable plasticity of dWAT, exhibiting its capacity for reversible dedifferentiation in murine models (51, 52). In the skin, dWAT serves as a critical site for recruiting immune cells such as T cells and macrophages, which release pro-inflammatory cytokines (51, 52). Hence, the diminished expression of inflammatory cytokines observed in the skin of CR-fed mice could be attributed to adipose hypotrophy. However, the implications of dWAT reduction induced by CR on skin homeostasis warrant further investigation to fully comprehend the effects.

Preclinical and clinical studies over the past years have demonstrated that the psoriasis is associated with changes of calorie intake, and our investigation sought to explore the impact of CR on hyperplasic disease development (53). Our findings revealed that CR exerted a protective effect against the development of psoriatic phenotypes in an IMQ-treated mouse model of psoriasis. We found that CR led to a reduction in the infiltration of immune cells such as macrophages and T cells into the skin. Additionally, there was a decrease in the expression of pro-inflammatory cytokines TNF-*α* and IFN-*γ* following 2 weeks of CR. IL-17 signaling pathway plays a crucial role in the pathogenesis of psoriasis (54, 55). IL-17A mRNA level was comparable between IMQ-treated, CR-fed mouse skin and AL-fed mouse skin. Intriguingly, compared to IMQ-treated AL mice, both IL-17A and IL-17RA expression at protein level were decreased in IMQ-treated CR mouse skin, potentially contributing to the reduced psoriatic phenotypes observed with CR. Unexpectedly, IL-23A mRNA level was slightly increased in IMQ-treated CR-fed mouse skin compared with IMQ-treated AL-fed mouse skin. This could be attributed to other cell types contributing to IL-23A production. Recent research has suggested that diet-induced dysbiosis may trigger IL-23 expression, adding complexity to our understanding (56, 57).

CR treatment attenuated the pathogenesis of psoriasis phenotypes. This amelioration of psoriasis-like symptoms in CR mice was accompanied by a reduction in mTOR signaling activation in psoriatic skin (30, 46). mTOR is a central hub of nutrient signaling (58) and plays an important role in skin homeostasis, repair and the pathogenesis of skin diseases (46, 59). Particularly, several studies have demonstrated that mTORC1 signaling activity is induced in psoriatic skin and its aberrant activation contributes to the production of important proinflammatory cytokines and chemokines in keratinocytes. These cytokines and chemokines further recruit immune cells to the skin, leading to development of psoriatic pathogenesis (49, 60, 61). In our study, we observed a decreased mTORC1 activation in the psoriatic skin lesions of CR-treated mice. This suggests that CR inhibits keratinocyte proliferation, differentiation, and mitigates the inflammatory responses, potentially mediated by suppressing the mTORC1 signaling pathway. Due to a technical issue, we were unable to determine the quantity of S6 and 4E-BP1 total proteins in the skin tissues. Future investigation into the levels of phosphorylated-and total protein abundance may provide detailed mechanistic insights into the observed effects of CR in inhibiting IMQ-induced skin hyperplasia.

## 5 Conclusion

Taken together, our studies have revealed that even short-term CR interventions can exert profound impacts on the structure and function of the skin. Specifically, CR has demonstrated potential as a non-pharmacological approach to attenuating skin inflammatory responses, reducing the development of hyperplastic skin diseases, and preserving the integrity and function of the skin barrier. However, several limitations should be noted in our study, including the lack of uniform standards for the duration of dietary restriction, the specific nutrients restricted (e.g., sugar, fat, protein), and the degree of restriction. Additionally, differences in physiological metabolism and genetic factors across species, as well as challenges in precisely controlling food intake and feeding times in animal experiments, may lead to varying conclusions. In the future, addressing these factors and developing safer and more effective methods, for example long-term CR without malnutrition, will be crucial for translating the findings of CR effects into human applications.

## Data Availability

The datasets presented in this study can be found in online repositories. The names of the repository/repositories and accession number(s) can be found in the article/Supplementary material.

## Supplementary material

SUPPLEMENTARY FIGURE S1 CR weakens skin barrier proteins expression in IMQ-induced epidermal hyperplasia. **(A)** Left, K14 staining of skin lesions by the immunofluorescence assay. Right, the mean fluorescence intensity of K14 expression in the skin lesions. **(B)** Left, K10 staining of skin lesions by the immunofluorescence assay. Right, the mean fluorescence intensity of K10 expression in the skin lesions. **(C)** Left, Filaggrin staining of skin lesions by the immunofluorescence assay. Right, the mean fluorescence intensity of Filaggrin expression in the skin lesions. **(D)** Left, Loricrin staining of skin lesions by the immunofluorescence assay. Right, the mean fluorescence intensity of Loricrin expression in the skin lesions. Scale bar: 100 μm. Each dot represents one mouse. Data are presented as mean ± SEM (*n*=3), and statistical significance was determined by two-way ANOVA for multiple comparisons. * *p* < 0.05, ** *p* < 0.01, *** *p* < 0.001; ns, not significant; e, epidermis; d, dermis.
