# Supplementary figures and images for: Caloric restriction impacts skin barrier function and attenuates the development of hyperplasia skin disease
Source: Front Nutr. 2024 Sep 20;11:1423524. doi: 10.3389/fnut.2024.1423524 (PMC11449767; doi:10.3389/fnut.2024.1423524)

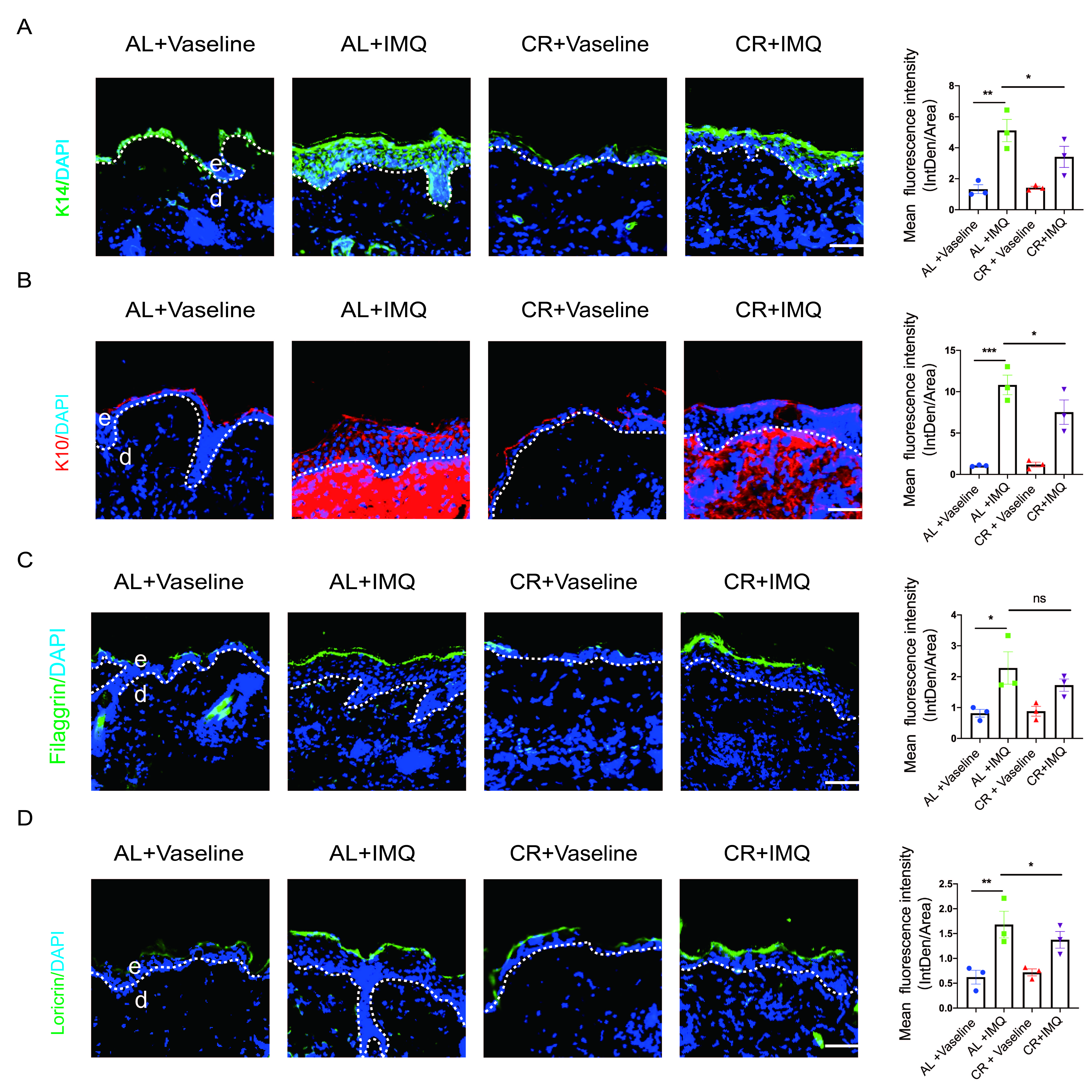

Supplement: SUPPLEMENTARY FIGURE S1 — CR weakens skin barrier proteins expression in IMQ-induced epidermal hyperplasia. (A) Left, K14 staining of skin lesions by the immunofluorescence assay. Right, the mean fluorescence intensity of K14 expression in the skin lesions. (B) Left, K10 staining of skin lesions by the immunofluorescence assay. Right, the mean fluorescence intensity of K10 expression in the skin lesions. (C) Left, Filaggrin staining of skin lesions by the immunofluorescence assay. Right, the mean fluorescence intensity of Filaggrin expression in the skin lesions. (D) Left, Loricrin staining of skin lesions by the immunofluorescence assay. Right, the mean fluorescence intensity of Loricrin expression in the skin lesions. Scale bar: 100 μm. Each dot represents one mouse. Data are presented as mean ± SEM (n=3), and statistical significance was determined by two-way ANOVA for multiple comparisons. * p < 0.05, ** p < 0.01, *** p < 0.001; ns, not significant; e, epidermis; d, dermis. [file Image_1.JPEG]
